# Supplementary material for: Multivalency ensures persistence of a +TIP body at specialized microtubule ends
Source: Nat Cell Biol. 2022 Dec 19;25(1):56–67. doi: 10.1038/s41556-022-01035-2 (PMC9859758; doi:10.1038/s41556-022-01035-2)
Supplement: Supplementary file 1 — Reporting Summary [file 41556_2022_1035_MOESM1_ESM.pdf]

nature portfolio

Corresponding author(s): Yves Barral

Last updated by author(s): Aug 17, 2022

Reporting Summary

Nature Portfolio wishes to improve the reproducibility of the work that we publish. This form provides structure for consistency and transparency in reporting. For further information on Nature Portfolio policies, see our Editorial Policies and the Editorial Policy Checklist.

Statistics

For all statistical analyses, confirm that the following items are present in the figure legend, table legend, main text, or Methods section.

n/a	Confirmed	
☐	☒	The exact sample size (*n*) for each experimental group/condition, given as a discrete number and unit of measurement
☐	☒	A statement on whether measurements were taken from distinct samples or whether the same sample was measured repeatedly
☐	☒	The statistical test(s) used AND whether they are one- or two-sided<br>*Only common tests should be described solely by name; describe more complex techniques in the Methods section.*
☒	☐	A description of all covariates tested
☐	☒	A description of any assumptions or corrections, such as tests of normality and adjustment for multiple comparisons
☐	☒	A full description of the statistical parameters including central tendency (e.g. means) or other basic estimates (e.g. regression coefficient) AND variation (e.g. standard deviation) or associated estimates of uncertainty (e.g. confidence intervals)
☐	☒	For null hypothesis testing, the test statistic (e.g. *F*, *t*, *r*) with confidence intervals, effect sizes, degrees of freedom and *P* value noted<br>*Give P values as exact values whenever suitable.*
☒	☐	For Bayesian analysis, information on the choice of priors and Markov chain Monte Carlo settings
☒	☐	For hierarchical and complex designs, identification of the appropriate level for tests and full reporting of outcomes
☒	☐	Estimates of effect sizes (e.g. Cohen's *d*, Pearson's *r*), indicating how they were calculated

Our web collection on statistics for biologists contains articles on many of the points above.

Software and code

Policy information about availability of computer code

Data collection	For data collection, the commercial software of the microscope manufacturers was used. Specifically, softWoRx 6.5.2 was used for collection on Personal DeltaVision, VisiView 5 was used for collection on Visitron Spinning Disk. ASTRA 6 was used to acquire elution profiles during MALS erperiments.
Data analysis	For data analysis, Fiji 2.3.0/ImageJ 1.53 was used for image analysis, Excel 16 and Matlab R2019b were used for calculations and statistics, Graphpad Prism 9 was used for plots and statistics, ASTRA 6 was used to estimate molecular weights in MALS experiments, Global3 was used to interpret CD spectra, Pymol 2.5 was used for to create structure representations, CLC Genomics Workbench was used to create sequence alignments.

For manuscripts utilizing custom algorithms or software that are central to the research but not yet described in published literature, software must be made available to editors and reviewers. We strongly encourage code deposition in a community repository (e.g. GitHub). See the Nature Portfolio guidelines for submitting code & software for further information.

Data

Policy information about availability of data

All manuscripts must include a data availability statement. This statement should provide the following information, where applicable:

- Accession codes, unique identifiers, or web links for publicly available datasets
- A description of any restrictions on data availability
- For clinical datasets or third party data, please ensure that the statement adheres to our policy

All data and any additional information required to analyze the data reported in this paper are available from the lead contact upon request. The previously published crystal structure of Naumovozyma Castellii Kar9 N-terminal domain is available under PDB ID 7AG9.

Field-specific reporting

Please select the one below that is the best fit for your research. If you are not sure, read the appropriate sections before making your selection.

☒ Life sciences ☐ Behavioural & social sciences ☐ Ecological, evolutionary & environmental sciences

For a reference copy of the document with all sections, see nature.com/documents/nr-reporting-summary-flat.pdf

Life sciences study design

All studies must disclose on these points even when the disclosure is negative.

Sample size	No statistical method was used to predetermine sample size. Sample size (cell number) was chosen based on effect size and variance within the sample. We systematically analyzed all cells in the correct cell cycle stage at least until statistical significance was reached for clear phenotypes with importance to the manuscript. For most experiments, the observed differences in phenotypes were large. Therefore even 50 cells were often more than enough.
Data exclusions	No data were excluded from the analyses.
Replication	For most live-cell microscopy data, biological duplicates or triplicates were used and images were acquired on multiple days (technical replicates). The only exception is the tip tracking assay, which is very demanding in terms of data analysis. There, only 1 clone per strain was analyzed. Due to high protein consumption, phase separation experiments were not systematically performed with replication (replication showed no significant differences where it was performed).
Randomization	The experiments were not randomized. In this study, the phenotypes of pure clones with known engineered genotypes were compared. Thus, no experiments required randomization to eliminate confounding effects.
Blinding	The investigators were not blinded for data analysis. The mutant strains were often discernible by eye compared to the wild type control, due to the very obvious differences in the phenotypes. Thus, blinding was not truly feasible.

Reporting for specific materials, systems and methods

We require information from authors about some types of materials, experimental systems and methods used in many studies. Here, indicate whether each material, system or method listed is relevant to your study. If you are not sure if a list item applies to your research, read the appropriate section before selecting a response.

Materials & experimental systems

n/a	Involved in the study	
☒	☐	Antibodies
☒	☐	Eukaryotic cell lines
☒	☐	Palaeontology and archaeology
☒	☐	Animals and other organisms
☒	☐	Human research participants
☒	☐	Clinical data
☒	☐	Dual use research of concern

Methods

n/a	Involved in the study	
☒	☐	ChIP-seq
☒	☐	Flow cytometry
☒	☐	MRI-based neuroimaging
