# Supplementary figures and images for: Multivalency ensures persistence of a +TIP body at specialized microtubule ends
Source: Nat Cell Biol. 2022 Dec 19;25(1):56–67. doi: 10.1038/s41556-022-01035-2 (PMC9859758; doi:10.1038/s41556-022-01035-2)

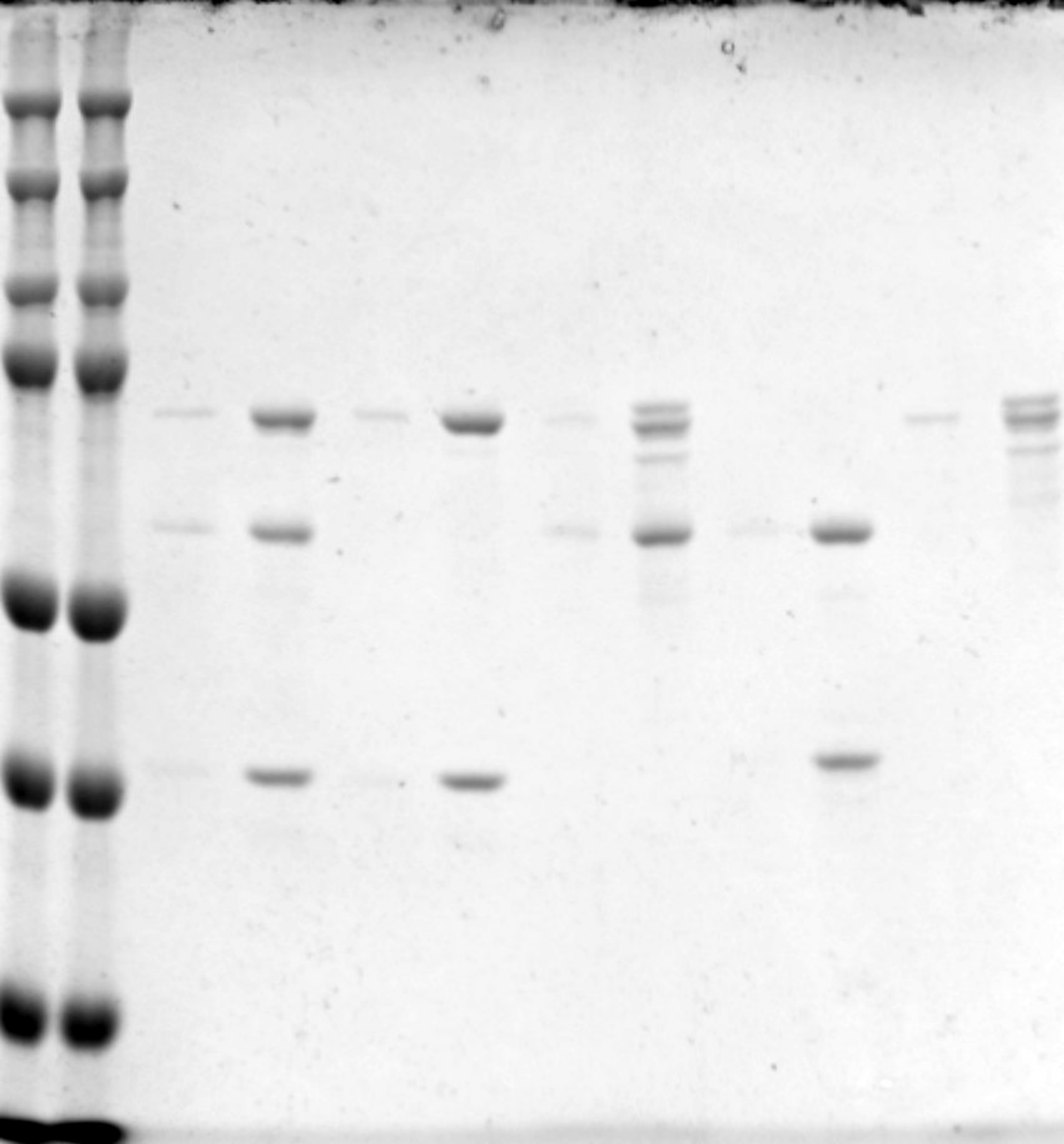

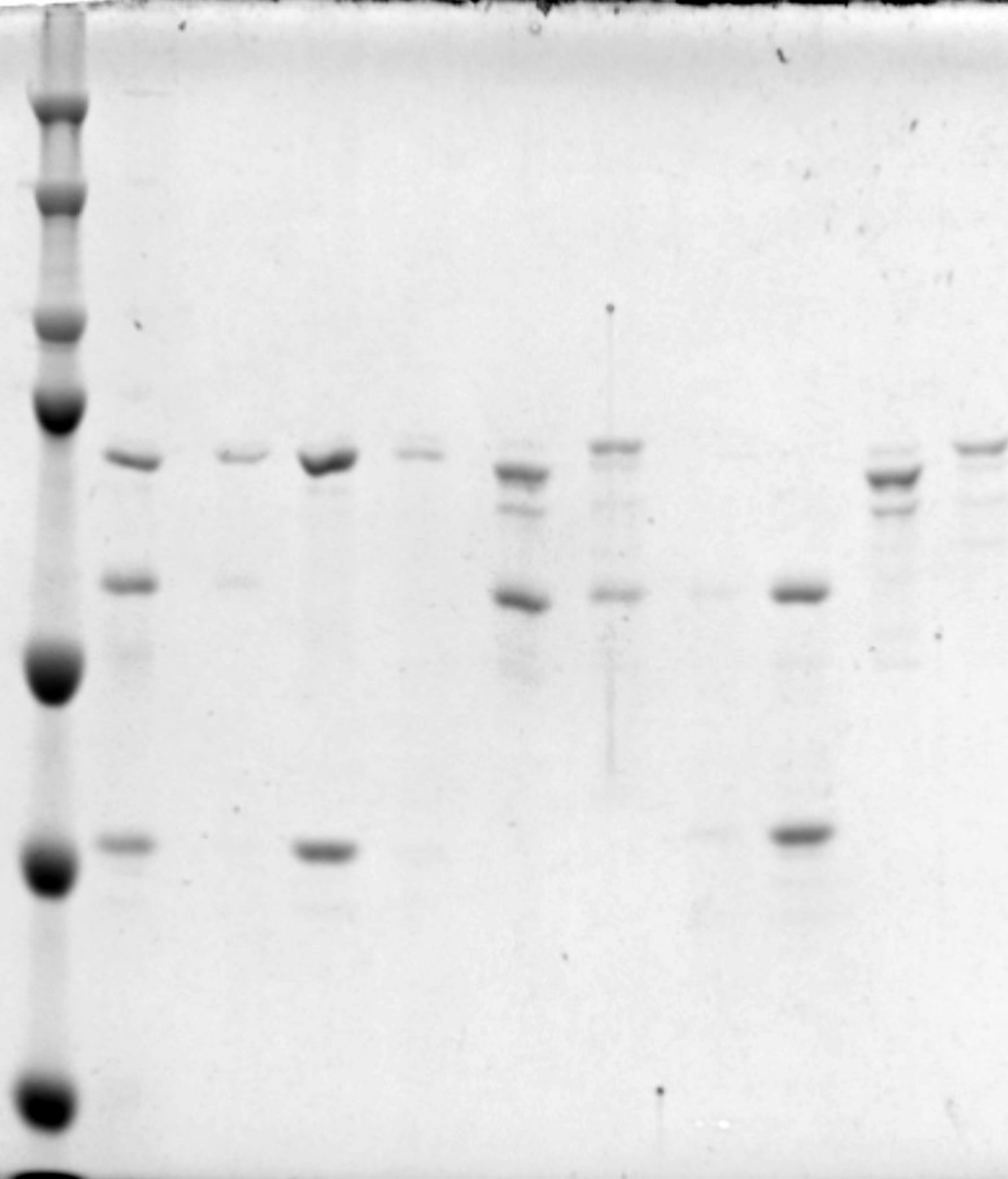

Supplement: Source Data Fig. 1 — Unprocessed gels Fig. 1. [file 41556_2022_1035_MOESM14_ESM.pdf]
